# A cloaked glutamate decarboxylase sustains the GABA shunt in *Mycobacterium tuberculosis*

**DOI:** 10.1073/pnas.2619778123

**Published:** 2026-08-19

**Authors:** H. Minh Thai, Debbie M. Hunt, Yugen Miyahara, Manisha Priya, Htin L. Aung, Rémi Zallot, Luiz Pedro S. de Carvalho

**Affiliations:** 1 Department of Chemistry, The Herbert Wertheim UF Scripps Institute for Biomedical Innovation and Technology, Jupiter, FL 33458, United States; 2 The Skaggs Graduate School, The Scripps Research Institute, La Jolla, CA 92037, United States; 3 USA Mycobacterial Metabolism and Antibiotic Research Laboratory, The Francis Crick Institute, London NW1 1AT, United Kingdom; 4 Department of Microbiology and Immunology, Faculty of Biomedical Sciences, Division of Health Sciences, University of Otago. PO Box 56, Dunedin 9016, New Zealand; 5 Department of Life Sciences, Manchester Metropolitan University. Dalton Building, Manchester M1 5GD, United Kingdom

## Abstract

Despite the exponential growth in genome sequencing, the functional annotation of genes, particularly the discovery of novel enzymatic activities, remains a formidable challenge. This gap is exacerbated by annotation biases that propagate assumptions about enzyme function across homologous sequences. Here, we report the discovery of a previously unrecognized enzymatic activity within a presumed well-characterized enzyme family. The gene *rv2531*c, under strong purifying selection across *Mycobacterium tuberculosis* strains, has been annotated as a member of the lysine-ornithine-arginine (KOR) decarboxylase superfamily, which includes over 26,000 sequences. Contrary to this annotation, we show that Rv2531c does not decarboxylate KOR substrates. Instead, using an integrative approach combining bioinformatics, microbiology, metabolomics, and enzymology, we demonstrate that Rv2531c is a novel L-glutamate decarboxylase that sustains carbon flux through the GABA shunt in *M. tuberculosis*. This newly identified subfamily of enzymes is conserved across Bacteria, Archaea, and Eukarya, and exhibits distinct structural and kinetic features, including an additional domain, hysteresis, and strong positive cooperativity. These characteristics differentiate it from canonical, enterobacterial KOR decarboxylases. More broadly, our findings challenge the narrow substrate and functional scope traditionally assigned to the KOR-DC superfamily. We propose that many members of this large and diverse enzyme family catalyze distinct reactions and participate in previously unrecognized metabolic pathways, revealing a broader and more nuanced role for PLP-dependent enzymes in microbial physiology and evolution.

## Introduction

The post-genome era, marked by the sequencing of thousands of genomes, has presented both opportunities and challenges in the field of enzymology. While vast amounts of genomic data are now available, facilitating the annotation of known enzyme activities, discovering novel enzymatic functions remains a daunting task. Most enzymes are routinely annotated based solely on sequence homology to known proteins, which can be misleading due to functional divergence among homologs (1, 2). Even highly similar sequences can perform distinct catalytic functions due to subtle differences in active site composition and substrate specificity (3). Sequence identity-only annotations are highly error-prone below 30% threshold (4). Moreover, enzyme promiscuity, the ability of an enzyme to catalyze side reactions, further complicates function prediction (5). Such promiscuity can lead to misannotation, as traditional sequence-based approaches struggle to account for multiple functionalities within the same protein family (6). Additionally, having a structure or computational model of a particular enzyme does not necessarily help beyond what primary sequence and fold predictions already forecast. That is, structural similarity does not always correlate with functional similarity, as enzymes with nearly identical folds can catalyze widely different reactions, with rates spanning multiple orders of magnitude (7). While misannotation of enzyme function remains a critical challenge in genomics, these “cloaked” enzymes represent an untaped source for the discovery of novel chemistry and biology. For example, Erb and colleagues have discovered that a RuBisCO homolog from *Rhodospirillum rubrum* is in fact a 5-methylthio-D-ribulose-1-phosphate isomerase, involved in S-adenosylmethionine and isoprenoid metabolism and not in carbon fixation (8). Peck et al. have identified a previously unknown glycyl radical enzyme from *Bilophila wadsworthia* involved in taurine catabolism, producing H_2_S (9). We have discovered a bifunctional (*R*)-3-hydroxy-3-methylglutaryl-/(*S*)-citramalyl-CoA lyase, previously annotated as a citryl-ACP lyase in *Mycobacterium tuberculosis* (10). Common to these three studies, metabolomics has emerged as promising method for *de novo* enzyme function discovery, with methods such as activity-based metabolomic profiling and global metabolomic profiling enabling the identification of novel enzymatic activities in complex biological systems and samples (11, 12). These approaches synergize with traditional computational, genomics-based and enzymological methods by providing empirical evidence of enzyme function when pure enzyme or an organism with a gene deletion or knockdown can be investigated.

L-Lysine decarboxylase (LDC), L-ornithine decarboxylase (ODC), and L-arginine decarboxylase (ADC), collectively referred to as KOR decarboxylases, are key enzymes involved in polyamine and amino acid metabolism in all domains of life. These type II, pyridoxal 5’-phosphate (PLP)-dependent enzymes (13) catalyze the removal of carboxyl groups from their respective amino acid substrates - ornithine, arginine, and lysine - yielding putrescine, agmatine, and cadaverine, respectively. Polyamines play critical roles in bacteria, including cell growth and proliferation, biofilm formation, acid resistance, antibiotic sensitivity, oxidative stress resistance, gene expression and protein synthesis (14–16). Naturally, the homologs from the model bacterium *Escherichia coli* have been extensively studied, including their biological, enzymological and structural aspects (17–21). In fact, *E. coli* contains six homologs of KOR decarboxylases (AdiA, SpeC, SpeF, SpeA, CadA and LdcC) (19). Despite that, little is known about these enzymes and their substrate specificity beyond enterobacteria. For example, in *M. tuberculosis*, the etiologic agent of human tuberculosis, a single KOR decarboxylase homolog exists, encoded by *rv2531*c gene (UNIPROT ID I6X4K0). Curiously, the observed Rv2531c protein is significantly longer than the *E. coli* KOR decarboxylases by approximately 300 residues (22, 23). Furthermore, the *rv2531*c gene has been annotated as non-essential in multiple transposon saturation studies (24, 25), which is at odds with its putative function as the sole producer of essential polyamines such as putrescine and cadaverine, in *M. tuberculosis*. Finally, the primary amino acid identity between the *E. coli* KOR decarboxylases and Rv2531c is less than 30%, indicating poor primary sequence conservation. Together, these observations cast doubt on the functional annotation of Rv2531c as a putative KOR decarboxylase and suggest Rv2531c might have distinct enzymatic and biological functions in *M. tuberculosis* and in other organisms.

In this study we elucidated the biochemical and metabolic functions of Rv2531c. Our results reveal that the KOR decarboxylase superfamily is far more diverse than originally thought and that it is composed of three distinct types of enzymes, with different domain organizations, and at least two unique biochemical activities. We show that Rv2531c is not a KOR decarboxylase and reveal a new function in the GABA shunt in *M. tuberculosis* and likely in many other bacteria and eukaryotes.

## Results

### Rv2531c belongs to a previously unknown, longer form of KOR decarboxylases

**Figure 1:**
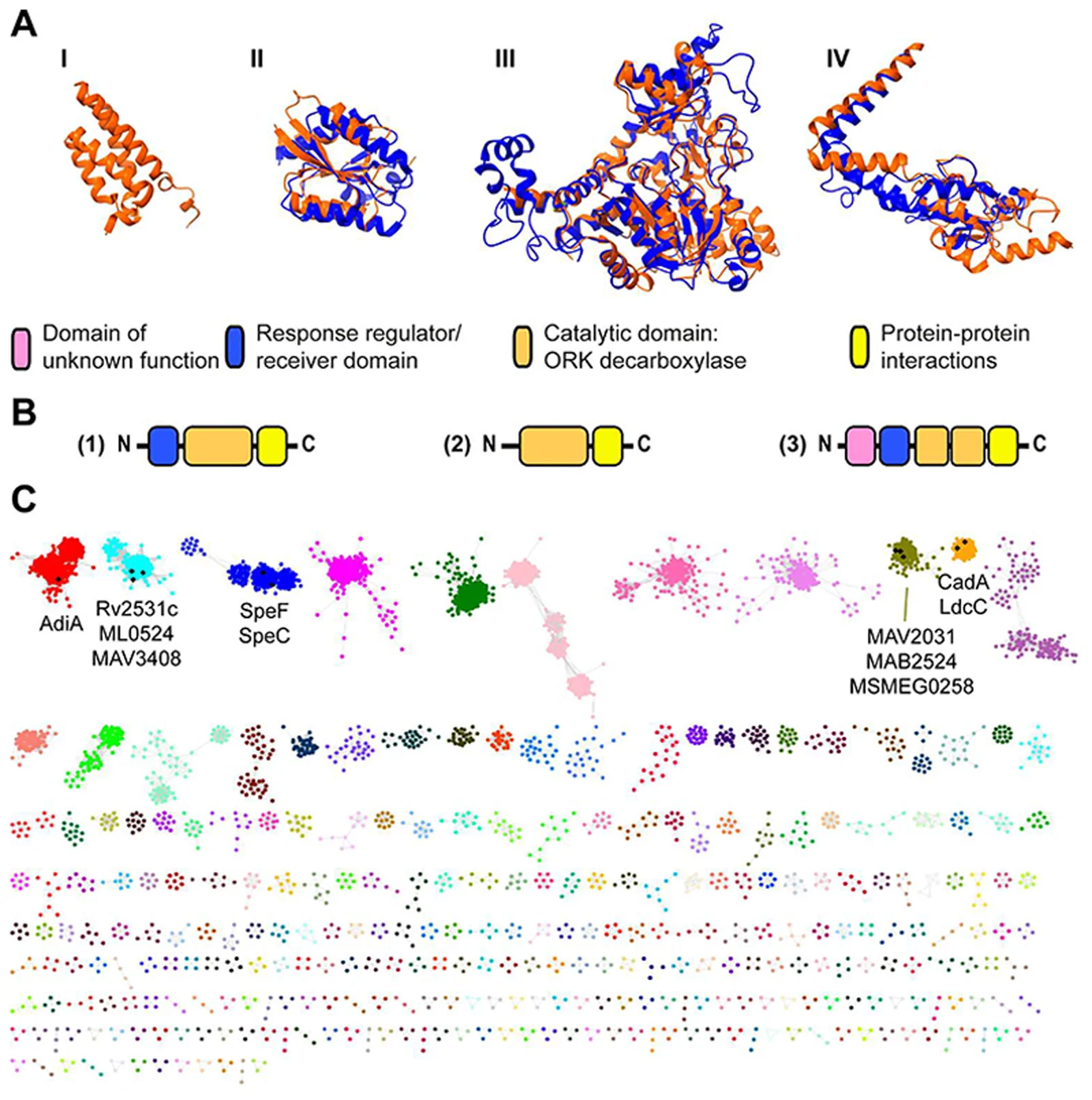
Structure, domain organization and sequence similarity network of Rv2531c homologs in bacteria. (**A**) Superimposition of the domains of AdiA (PDB_ID 2VYC) in blue and of the PLP-bound Rv2531c (PDB_ID 9N0O), in orange. RMSD ~ 0.9-1.4. I, additional domain not found on AdiA, II, response receiver domain, III, catalytic domain, and IV, C-terminal domain. Predicted functions of each domain. **(B)** Domain organization of homologs. (**C**) Partial UniRef90 SSN for members of the IPR000310 family, with an alignment score threshold of 185. Cluster containing only singletons or two sequences have been removed for clarity. Full SSN shown in Fig. S1. Selected *Mycobacterium* species and *E. coli* homologs are shown as black diamonds.

Using the primary amino sequence of Rv2531c’s catalytic domain, we employed BLAST (26) to retrieve the top homologous protein sequences in NCBI. As expected, based on its annotation, we retrieved homologs proteins such as *E. coli* AdiA, SpeF, SpeC, SpeA, CadA and LdC, all of which are KOR DCs, and other mycobacterial homologs, such as *Mycobacterium leprae* ML0524, *Mycobacterium avium* MAV3408 and MAV2031, *Mycobacterium smegmatis* MSMEG0258 and *Mycobacterium abscessus* MAB2524. As shown in Fig. 1A, superimposition of the structure of *E. coli* AdiA and the PLP-loaded structure of Rv2531c (PDB_ID 9N0O) reveals clear differences. Rv2531c contains one extra domain, compared to AdiA. Conversely, some homologs lack the N-terminal domain found in canonical KOR decarboxylases. Based on these results, we conclude that Rv2531c closest homologs exist in three distinct forms. The first group contains all the *E. coli* KOR decarboxylases, several of which have been experimentally characterized (17–21). These proteins are approximately 650 residues long and contain three domains (Fig. 1B), a response regulator domain N-terminal domain, a central catalytic domain, and a C-terminal domain likely involved in protein-protein interactions, found exclusively in KOR decarboxylases. Most KOR decarboxylase structures determined to date revealed a homo-decameric arrangement, with two pentameric rings (20, 21, 27–29). A second group contains several sequences of proteins that have not been characterized experimentally, these are shorter forms of the enzyme, with approximately 450 residues in length, and lack the response regulator domain (Fig. 1B). Rv2531c belongs to the third group (Fig. 1B), in which no enzymes have been experimentally characterized. Despite sequence similarities of less than 30% with other KOR decarboxylases, Rv2531c is longer than most homologs, with 947 residues in length. This longer sequence is due to an additional domain, located at the N-terminus (Fig. 1B). No function has been attributed to this domain, and therefore we have labeled it a domain-of-unknown-function (DUF). In summary, KOR decarboxylases exist in three unique forms, a long form (including Rv2531c and its homologs in *M. leprae* and *M. avium*), an intermediate form (including all *E. coli* KOR decarboxylases) and a short form (including homologs from *M. abscessus, M. smegmatis* and *M. avium*).

To understand the distribution and the diversity of these enzymes in nature, we calculated a sequence similarity network (SSN) of the InterPro family IPR000310, which Rv2531c belongs to (27, 30). Fig. 1C illustrates the UniRef90 SSN obtained. In this network, the 24,071 homologs are represented by 8,583 UniRef90 seed sequences as nodes. Members of the IPR000310 family are widespread in Bacteria but are also found in Archaea and Eukarya. The alignment score threshold applied (185) was defined as the lowest to separate the characterized arginine, lysine and ornithine decarboxylases into separate (presumed) isofunctional clusters. At this threshold, Rv2531c is located into the second largest UniRef90 cluster, containing 618 UniRef90 sequences representing 1,231 unique sequences. The Rv2531c cluster contains sequences from various Phyla, including Actinomycetes (e.g., *Mycobacterium* and *Arthrobacter* sp.), Alphaproteobacteria (e.g., *Rhizobium* species), Betaproteobacteria (e.g., *Burkholderia* species), Gammaproteobacteria (e.g., *Acinetobacter, Halomonas* sp.), Conoidasida (e.g., *Toxoplasma* sp.), Flavobacteriia (e.g., Flavobacterium, *Salegentibacter* and *Subsaximicrobium* sp.), Myxococcia (e.g., *Myxococcus, Enhygromyxa* sp.), and surprisingly also contains sequences from Eukaryotes, notably, *Plasmodium* species. Together, these results indicate that Rv2531c and its closest homologs represent a common form of the enzyme and are found in Bacteria and Eukarya.

We next tested whether Rv2531c is being selected during the evolution of *M. tuberculosis*. For this we calculated the non-synonymous over synonymous ratio (*dN/dS*), using *rv2531*c sequences from 4685 *M. tuberculosis* genomes. The *dN/dS* ratio is a key parameter in population genetics, estimating the evolution of DNA sequences. Ratios > 1 indicate Darwinian selection, ratios < 1 indicate purifying selection and a ratio = 1 no selection (neutral). The *dN/dS* ratio obtained is 0.23, which indicates *rv2531*c is under strong purifying (negative) selection, suggesting that deleterious mutations are been removed from the population, ensuring that Rv2531c maintains its function.

### Rv2531c is not a KOR decarboxylase

Rv2531c shares only 26% amino acid identity over 67% of its sequence with the well-characterized *E. coli* L-arginine decarboxylase (AdiA), and therefore, its function cannot be unambiguously established from primary sequence homology alone (4). To study the function of Rv2531c, we generated a clean genetic deletion of the *rv2531*c gene in *M. tuberculosis* H37Rv using a sequential crossover method (31), designated Δ*rv2531*c, and created a complemented strain (Δ*rv2531*c::*rv2531*c). Fig. S2 illustrates the disappearance of Rv2531c from protein extract obtained from the Δ*rv2531*c strain, compared to the parent and the complemented strains. This result also demonstrates that Rv2531c is constitutively expressed in *M. tuberculosis*. In agreement with transposon mutagenesis data (24, 25), the fact that we obtained a deletion mutant of *rv2531*c indicates that Rv2531c is not essential in *M. tuberculosis* grown in Middlebrook 7H9/7H10 media (Fig. S2).

**Fig. 2:**
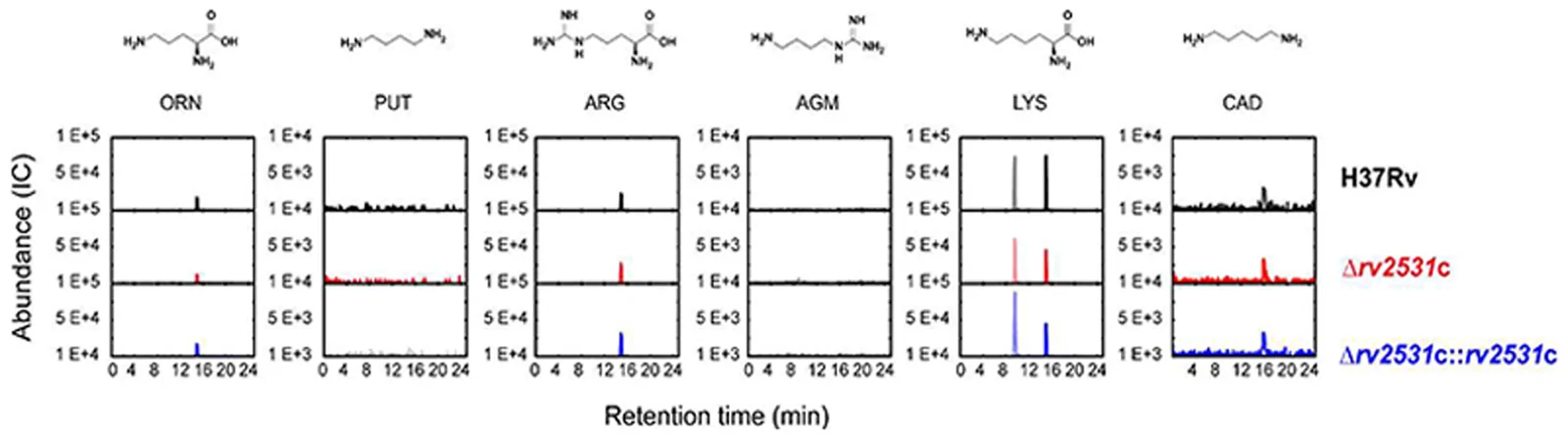
Rv2531c does not produce polyamines from basic amino acids. Metabolomics results for the amino acids ornithine, Orn (M+H)^+^ 133.0972; arginine, (M+H)^+^ 175.1190; and lysine, (M+H)^+^ 147.1128; and the polyamines putrescine, Put (M+H)^+^ 89.1073; agmatine, Agm (M+H)^+^ 131.1291; and cadaverine, Cad (M+H)^+^ 103.1230. Data shown is from one experiment, with three biological replicates, representative of three independent experiments. Light colors on the panels for lysine represent the isobaric metabolite glutamine ((M+H)^+^ 147.0764). Y-axis scale for polyamines has been increased 10-fold compared amino acids to better illustrate the presence of cadaverine and its lack of change upon *rv2531*c deletion. Peaks were identified and retention time was matched with standards (Table. S3)

Next, we used a liquid chromatography-mass spectrometry (LC-MS) metabolomics method (32, 33) to quantitatively assess basic amino acid levels and their corresponding decarboxylation products (polyamines) in these strains. We found that deletion of Rv2531c did not induce any discernible changes in the levels of basic amino acids (L-lysine, L-arginine, and L-ornithine) that was complemented (Fig. 2). In agreement with these results, while we could observe a minute peak for cadaverine (decarboxylated L-lysine), its abundance was not altered by deletion of Rv2531c (Fig. 2). Curiously, we did not observe a peak corresponding to putrescine and agmatine in extracts from the three strains (Fig. 2), indicating that these polyamines are either present at very low abundance or not produced in *M. tuberculosis*. Thus, Rv2531c is not a functional homolog of *E. coli* KOR decarboxylases. Instead, Rv2531c likely serves a distinct and unique metabolic role in *M. tuberculosis* and related species.

### Rv2531c is a novel glutamate decarboxylase working on the GABA shunt

**Figure 3:**
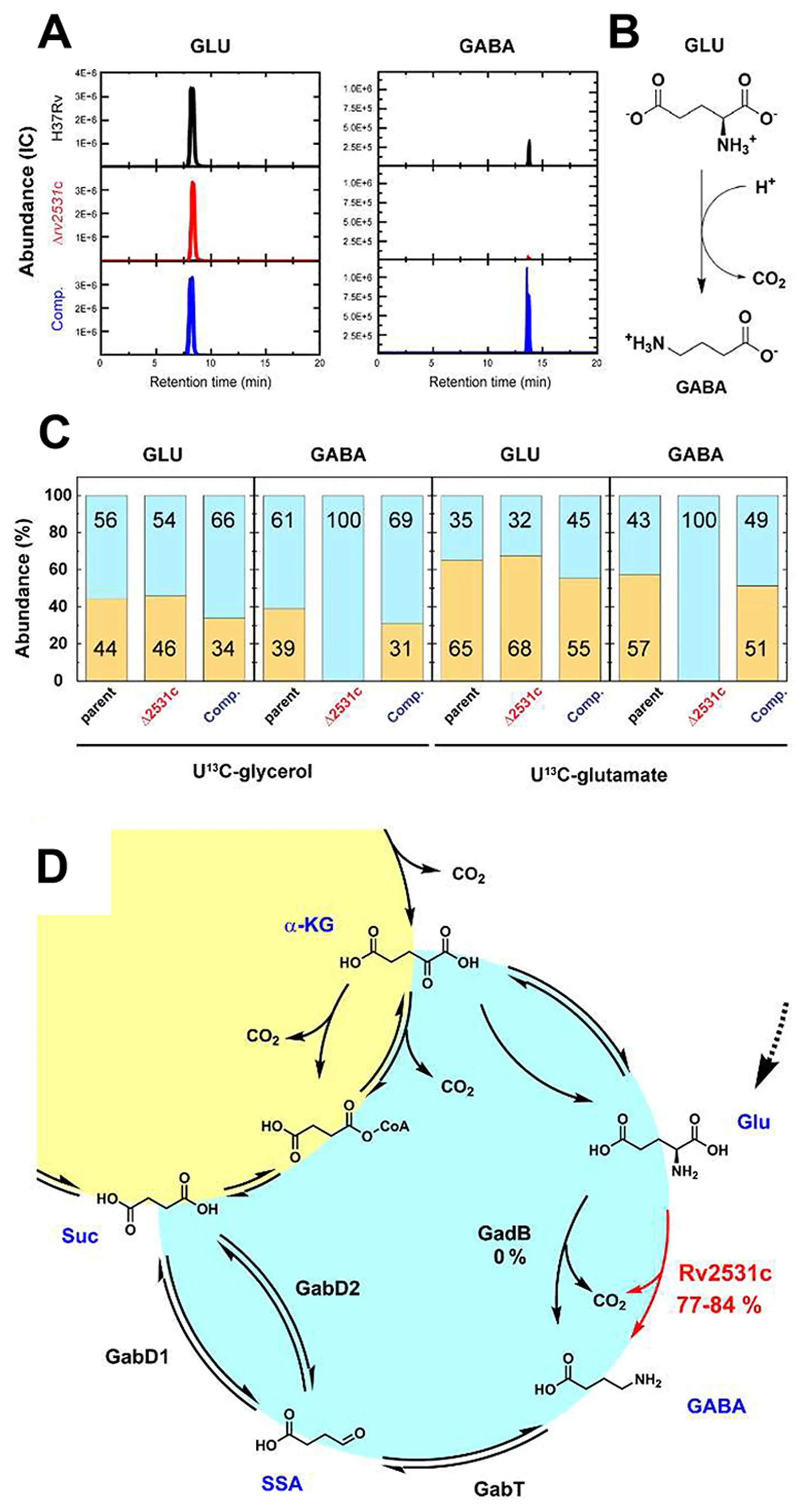
Rv2531c functions as the key glutamate decarboxylase in *M. tuberculosis*. (**A**) Metabolomics reveals disappearance of GABA in the Δ*rv2531*c strain. Data shown is from three biological replicates, representative of two independent experiments. (**B**) Glutamate decarboxylation reaction. (**C**) Stable isotope tracing of glutamate and GABA in parent, Δ*rv2531*c and Δ*rv2531*c::*rv2531*c strains expressed as %, with U-^13^C-glycerol and with U-^13^C-L-glutamate. Labeled pool is shown in orange and unlabeled pool is shown in light blue. (**D**) Schematic model of the GABA shunt (cyan) and the Krebs cycle (yellow) in *M. tuberculosis* H37Rv, with estimations of flux through GadB and Rv2531c, based on isotope tracing data (**C**). Some labels were omitted for clarity.

We hypothesized that Rv2531c possessed distinct substrate specificity compared to the *E. coli* KOR decarboxylases, as it can still be loaded with the PLP cofactor (Fig. S4). To identify its unknown substrate or product, we evaluated the decarboxylation products of other amino acids in our metabolomics data. Surprisingly, we find an 84% decrease in the level of γ-aminobutyrate (GABA) in our ΔRv2531c strain, shown in Fig. 3A. No reproducible and statistically significant changes were observed for other amino acids. The simplest route to GABA is the decarboxylation of L-glutamate (Fig. 3B). This change is exclusive to GABA, as no discernible alterations were observed with other amino acid products. The levels of glutamate did not change, as expected based on its important role in mycobacterial and Gram-positive bacterial physiology, as the main compatible osmolyte, counterion for K^+^, and also a co-substrate in most transamination reactions (34). These results indicate that despite its sequence and structural similarity to KOR decarboxylases, Rv2531c evolved to act on an acidic amino acid substrate instead of a basic one. This result positions Rv2531c in the GABA shunt, an anaplerotic route of the Krebs cycle, instead of polyamine biosynthesis (35).

To provide additional evidence for the role of Rv2531c as a bona fide L-glutamate decarboxylase in *M. tuberculosis*, and estimate its contribution to the GABA shunt, we employed stable isotope labelling in conjunction with metabolomics. We then tracked uptake of labeled U-^13^C-glycerol and U-^13^C-L-glutamate and their conversion into GABA, in the parent, Δ*rv2531*c and Δ*rv2531*c::*rv2531*c strains. With U-^13^C-glycerol, monitoring glutamate synthesis from α-ketoglutarate, we observe an average of 38% labeling of glutamate at 16h in the three strains (Fig. 3C). In contrast, no labeled GABA could be observed in the Δ*rv2531*c strain, while in the parent and complemented strains GABA labeled accounts for 31 - 39% of pool. With U-^13^C-L-glutamate, on average, 63% of the intrabacterial L-glutamate pool was labelled after 16 hours (Fig. 3C). Again, in sharp contrast, labelling of GABA was also not observed in the Δ*rv2531*c strain, despite being readily observed in the parent and complemented strains, varying between 51 and 57%. These results are consistent with the function of Rv2531c as a L-glutamate decarboxylase. Using our labeling data, we estimate that between 77 – 84% of glutamate decarboxylation in *M. tuberculosis* is performed by Rv2531c, with the remaining being generated by other enzymatic sources (Fig. 3D). As the no labeled GABA is observed in the Δ*rv2531*c strain, GadB (the canonical bacterial glutamate decarboxylase), which is present, is not decarboxylating L-glutamate. GadB is structurally unrelated to Rv2531c. Therefore, the newly discovered Rv2531c is not only a redundant enzyme, but it represents the dominant glutamate decarboxylase in *M. tuberculosis*.

### Reconstitution of Rv2531c-catalyzed glutamate decarboxylation

To provide direct and unequivocal evidence that Rv2531c is a glutamate decarboxylase, we expressed and purified Rv2531c from *E. coli*. Rv2531c was purified to homogeneity and shown to be in apo form (PLP-free – Fig. S4). As purified, Rv2531c elutes as a single symmetrical peak in gel filtration, with an elution volume consistent with a tetramer (Fig. S5), with an estimated molecular mass of 424 kDa, based on its primary sequence.

Unexpectedly, initial experiments aimed at determining activity of Rv2531c in the presence of PLP failed to show any activity utilizing L-glutamate, or other amino acids as substrates (not shown), suggesting that an essential cofactor was missing. Upon incubation of the enzyme with MgCl_2_, clear activity was observed (Fig. S6), indicating that Mg^2+^ ions are required for Rv2531c activity, a rare but precedented requirement for a PLP-dependent amino acid decarboxylases (36). Henceforth, all experiments with recombinant Rv2531c were performed in the presence of 10 mM MgCl_2_.

**Figure 4:**
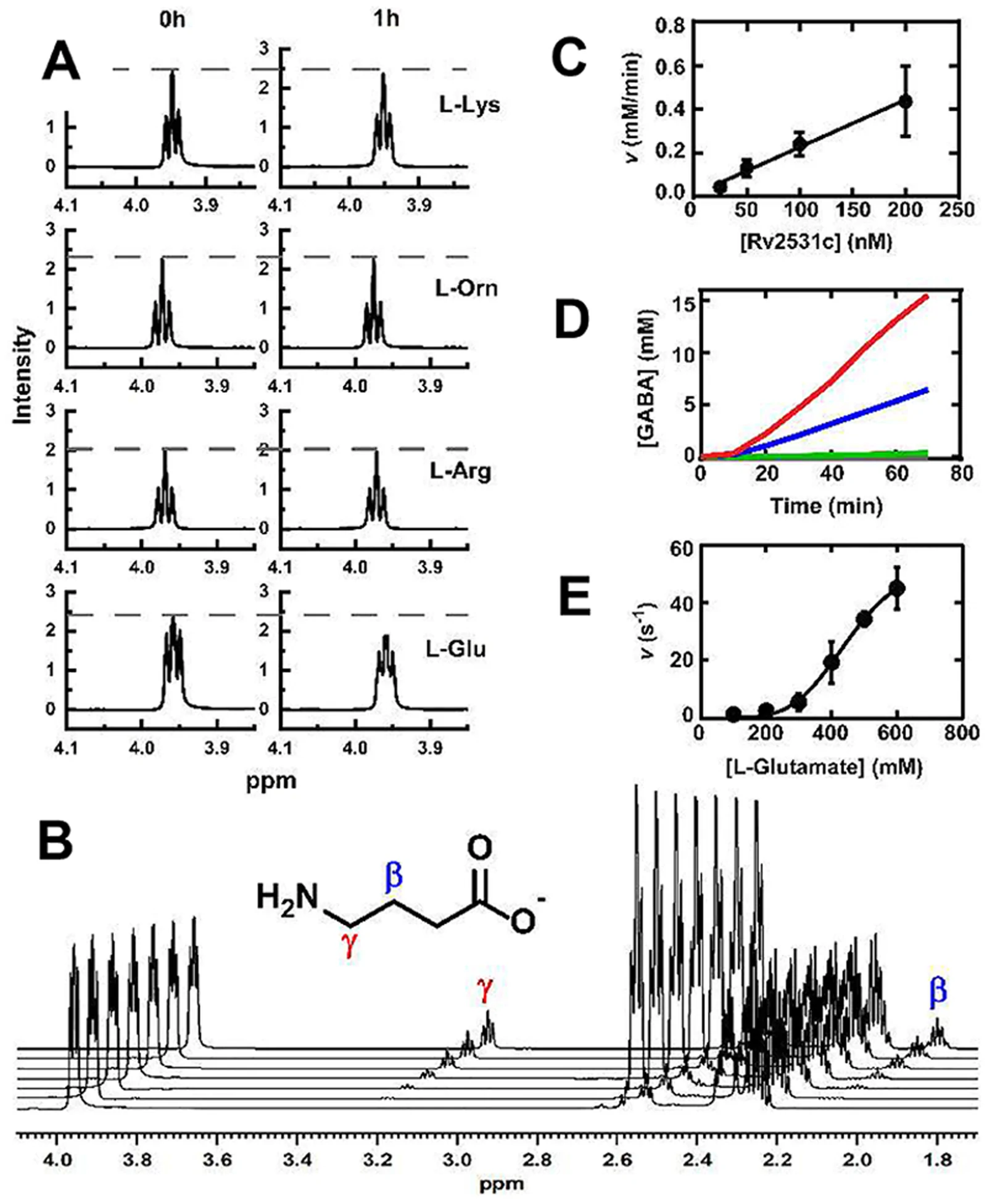
Unusual Rv2531c-catalyzed glutamate decarboxylation. All experiments were performed using ^1^H-NMR spectroscopy, in pH 7.3 sodium phosphate buffer containing 10 mM MgCl_2_, at 37°C. (**A**) Qualitative analysis of decarboxylation of amino acids by Rv2531c, monitoring the protons attached to the γ carbon, after 1 h reaction. (**B**) Time course of L-glutamate decarboxylation by Rv2531c at 100 mM glutamate. Each spectrum is the average of 64 scans, obtained every 10 min. (**C**) Glutamate decarboxylation is strictly and linearly dependent on enzyme concentration. Symbols are average of three independent experiments and line is the best fit to the simple linear regression model. (**D**) Exemplary time courses for L-glutamate decarboxylation show a significant lag. Each line corresponds to a different concentration of L-glutamate (green = 200, blue = 400 and red = 600 mM). (**E**) Plotting the initial rates from (**D**) versus L-glutamate concentration. Symbols are average of data obtained in three independent experiments and line is the fit to eq. 2 with an *n*^H^ = 4.

To test its catalytic activity, Rv2531c was incubated with L-lysine, L-ornithine, L-arginine, or L-glutamate, and the reaction progress was monitored using ^1^H nuclear magnetic resonance (NMR) spectroscopy. After one hour incubation, the α-carbon proton signal (triplet at 3.5 ppm) belonging to L-glutamate exhibited a decrease in intensity, while the corresponding signal from the other substrates remained unchanged (Fig. 4A). This result indicates that Rv2531c selectively catalyzes the decarboxylation of L-glutamate, corroborating our results from metabolomics. Analysis of the whole spectra shows new resonances corresponding to the β- and γ-methylene protons of γ-aminobutyric acid (GABA) appearing, confirming the enzymatic conversion of L-glutamate to GABA (Fig. 4B). The progressive increase in these signals over time further supports the catalytic activity of Rv2531c (Fig. 4B). To determine if decarboxylation was enzymatic or an artifact due to free PLP, the rate of L-glutamate decarboxylation was measured at varying Rv2531c concentrations (Fig. 4C). A linear correlation with Rv2531c concentration was observed, indicating that the reaction rate is directly proportional to enzyme concentration. Kinetic analysis was performed by measuring L-glutamate decarboxylation at increasing substrate concentrations (Fig. 4D). The Rv2531c time-courses show a pronounced lag, lasting at least 10 minutes at the highest L-glutamate concentration tested. This implies the enzyme is activating over time, also known as hysteresis (37, 38), and usually associated with a slow transition from an inhibited to an active form of an enzyme. The initial rates were taken from the linear region of the curves and plotted versus L-glutamate concentration (Fig. 4E). Inspection of the data indicated clear sigmoidal behavior instead of hyperbolic, and therefore the data were fitted to the Hill version of the Michaelis-Menten equation (Fig. 4E). Initial fitting of the data to the Hill equation (1) returned a Hill number of 4, which led us to model the kinetic behavior of Rv2531c assuming *n*^H^ = 4. The following kinetic parameters were obtained from the fit, *k*_cat_ = 69 ± 6 s^-1^, *K*_0.5_ = 509 ± 38 mM, *k*_cat_/*K*_0.5_ = 136 ± 16 M^-1^s^-1^. Together these results demonstrate that Rv2531c is a *bona fide* L-glutamate decarboxylase, which displays a relatively high *K*_0.5_, hysteresis and very strong positive cooperativity (*n*^H^= 4).

### The active site of Rv2531c: from KOR to glutamate decarboxylation

To investigate the molecular basis for the substrate specificity of Rv2531c, its preference for L-glutamate over basic amino acids, we compared the active site architecture of PLP-bound Rv2531c (PDB 9N0O) (39) with that of the inhibitor-PLP adduct of L-lysine decarboxylase from *Hafnia alvei* (PDB 9E0O) (40). For a more direct comparison, the ε-amino group of the L-α-hydrazino lysine analogue in 9E0O was computationally converted to a carboxylate, thereby mimicking the side chain of L-glutamate (Fig. S7A,B).

A key feature emerging from this comparison is a conserved lysine residue (K406) in Rv2531c. This residue is invariant among all available structural homologs and is positioned to form a salt bridge with the α-carboxylate of L-glutamate. K406 is optimally positioned to fine-tune the conformation and charge of H405, a residue positioned to form π-π interaction with the pyridine ring of PLP. The distance between K406 ε amino group and the imidazole ring from H405 is about 4.1 Å, slightly longer than a hydrogen bond (~ 3.5 Å) (41). At neutral pH and at this distance, K406 will be protonated and hence, H405 is unprotonated. This is important since a neutral imidazole is ideal for π-π interaction. Protonation of the imidazole would provide further electrostatic repulsion with the protonated K406 (42–45). The K406A variant cannot form the internal aldimine (Fig. S7C,D) and hence does not display enzymatic activity. The loss of function likely reflects multiple coupled roles: (i) direct stabilization of the PLP cofactor internal aldimine, through interaction with H405, (ii) stabilization of the substrate alpha carboxyl group, at the external aldimine stage and possibly during formation of the Michaelis complex, and (iii) reduced structural stability, as the K406A variant could not be concentrated to levels comparable to the wild-type enzyme.

Adjacent to K406, residue T606 from the adjacent protomer is positioned to hydrogen-bond with the α-carboxylate of L-glutamate. Although the modeled distance between T606 hydroxyl and L-glutamate α–carboxylate is also slightly longer than typical hydrogen-bonding upper limit (3.5 Å) (41), modest conformational adjustments within the active site could shorten this interaction. Consistent with a functional role, the T606V mutant remained soluble but displayed reduced stability and undetectable catalytic activity.

Inspection of the Rv2531c active site also revealed two arginine residues that are absent from the corresponding region of *H. alvei* LdcI (PDB 9E0O). These positively charged residues provide a plausible electrostatic complement to the carboxylate-containing side chain of L-glutamate. Modeling places R576 within salt-bridge distance of the substrate γ-carboxylate (Fig. S7B), suggesting a key role in substrate recognition. Additional residues, including R718 and N731, are positioned more distally in the model, but conformational flexibility during substrate binding or catalysis may allow transient interactions with the substrate. Mutational analysis revealed important roles for these residues on Rv2531c structural integrity. Variants R576A, R718A, and N731A all produced unstable proteins, preventing functional characterization. To test whether reversing the charge at position 576 might broaden substrate specificity toward basic amino acids, we generated the R576E mutant. However, this substitution also destabilized the protein, precluding a formal assessment of altered substrate preference.

Collectively, these structural and mutational results indicate that Rv2531c relies on a network of positively charged and hydrogen-bonding residues, centered around K406, T606, and R576, to selectively recognize and stabilize L-glutamate, instead of basic chain amino acids. The absence of analogous electrostatic features in lysine decarboxylases likely underlies the divergent substrate specificities of these enzyme families.

## Discussion

Sequence similarity networks reveal that most clusters of InterPro family IPR000310 do not contain any annotation. If we assume that clusters all have a different biological function (substrate specificity), there are still numerous functions to be identified, verified and annotated in this family. Furthermore, InterPro family IPR000310 contains sequences that can be divided into 48 distinct domain architectures, which indicates how versatile the amino acid decarboxylation core is and its potential for combination with other domain such as peptidases, hydrolases, phosphatases, deiminases, synthases and dehydrogenases. This new sub-family of PLP-dependent glutamate decarboxylases contains over 1,000 sequences, spread throughout various bacterial taxa and found in Eukaryotes such as *Plasmodium* species.

In this study we demonstrate that conserved *M. tuberculosis* protein Rv2531c belongs to a previously uncharacterized sub-family of PLP-dependent L-glutamate decarboxylases, previously mis-annotated as KOR decarboxylases. Despite a modest degree of sequence homology with the well-characterized ornithine/arginine/lysine (ORK) decarboxylases (17–21), Rv2531c acts on an acidic amino acid, instead of a basic one. Unlike previously studied KOR decarboxylases, which contain three domains, Rv2531c's additional N-terminal domain-of-unknown-function may play a role in the unique oligomeric state of this enzyme, a tetramer, compared to the previously characterized decameric versions (19–21), lacking this domain. Importantly, Rv2531c is under strong purifying selection, with *d*_N_/*d*_S_ ratio in the same range as the essential genes, further illustrating its importance for *M. tuberculosis*.

Our clean genetic deletion of the *rv2531*c gene in *M. tuberculosis* demonstrated that Rv2531c is not essential under standard culture conditions, in agreement with previous transposon mutagenesis data (24, 25). Yet, Rv2531c contributes significantly to the bacterium’s metabolism, as the dominant entry route for L-glutamate-derived carbon into the GABA shunt, an anaplerotic pathway of the Krebs cycle (35, 46). This finding underscores the enzyme's role in the maintenance of metabolic balance in *M. tuberculosis*, particularly under conditions where carbon source utilization might be sub-optimal (47–51) or in niches where L-glutamate is abundant. An important site of infection beyond the lung is the central nervous system, with brain tissue containing an average of 12 mM L-glutamate (52). It is likely that the GABA shunt is conditionally essential, that is, the GABA shunt is auxiliary to the Krebs cycle under many conditions (Fig. 3D), but dispensable under rich conditions such as in Middlebrook media. The observation that GadB does not substitute for Rv2531c’s absence, highlighted by the lack of labelled GABA in the Δ*rv2531*c strain (Fig. 3C) is surprising yet, it suggests that these enzymes are differentially regulated. This hypothesis is supported by chromatin immunoprecipitation data which shows that *gadB* is regulated by 4 transcription factors while Rv2531c is regulated by 10 transcription factors (53). From these regulators, only Rv0047c and Rv0967c are associated with both genes. Further studies are required to understand when the GABA shunt is essential for *M. tuberculosis* survival and to confirm or refute the function of GadB as an L-glutamate decarboxylase.

Enzymological experiments confirmed that Rv2531c functions as a L-glutamate decarboxylase, requiring magnesium ions for activity, a feature shared by few PLP-dependent enzymes (36). Rv2531c displays hysteresis and a high level of cooperativity, highlighting its conformational dynamics and regulatory potential, which might be uniquely positioned to serve bacterial metabolism (37, 54–57). In addition, recentwork from Izard group reveals that Rv2531c exists in an equilibrium between two states, an open and a closed state (39). This equilibrium might be responsible for the observed hysteresis and cooperative behaviors observed in our experiments. Further experiments are required to elucidate the physical basis for hysteresis and cooperativity in Rv2531c. Active site mutations revealed that nearly all residues altered lead to misfolded or unstable variants, likely because of the original residues requirement during conformational changes associated with catalysis. Nonetheless, our mutagenesis data implicates K406 as an important residue, required for internal aldimine formation, and perhaps for substrate binding.

Interestingly, Rv2531c’s activity was specific to L-glutamate, a substrate of particular importance in mycobacterial and Gram-positive bacterial physiology, both as a principal osmolyte and compatible solute, and as a precursor in various biosynthetic pathways (58–64). L-glutamate levels in *M. tuberculosis* are 100-fold above of the other amino acids under regular growth conditions. Importantly, the concentration of potassium L-glutamate in bacteria can rapidly reach 700-800 mM under osmotic stress (65, 66). Furthermore, even if the concentration of L-glutamate never saturates Rv2531c, and therefore it operates under *V*/*K* not *V*_max_ conditions, the physiologic impact of Rv2531c deletion was obvious, a ~ 90% decrease in the GABA pool size accompanied by a total shutdown of its synthesis from L-glutamate, illustrated by the complete disappearance of its labelling. To conclude, there are many of examples of other key metabolic enzymes that operate below saturation (in *V*/*K* conditions), for example multiple phosphatases, proteases, carbonic anhydrase and ammonia monooxygenase. In other words, *V*_max_ establishes the upper limit for catalysis by an enzyme, but its operating rate in cells is dependent on metabolism.

In summary, this study identifies Rv2531c as the principal L-glutamate decarboxylase in *M. tuberculosis*. We also demonstrate that 20% of GABA does not derive from L-glutamate or from α-ketoglutarate and therefore is not produced by an L-glutamate decarboxylase. Nonetheless, it is still possible that both enzymes are redundant, yet, under in vitro conditions used in these experiments GadB is fully repressed. Curiously, the GABA shunt’s final step in *M. tuberculosis* is catalyzed by GabD1 and GabD2 (67), strengthening the idea of the importance of this pathway. Future investigations into Rv2531c structure, regulation, inhibition, and broader implications in mycobacterial physiology are warranted to fully elucidate its role in *M. tuberculosis* lifestyle and pathogenesis. Our results refute the hypothesis that Rv2531c is a KOR decarboxylase and therefore indicate that another enzyme must exist to make cadaverine from L-lysine, which was visible in our metabolomic data. Beyond *M. tuberculosis*, homologs of Rv2531c, *i*.*e*. longer, four-domains-containing L-glutamate decarboxylases, are present in other pathogens, such as *M. avium* and *M. leprae*, and outside mycobacteria, in *Plasmodium* for example, demonstrating its potential role and importance in other organisms.

## Material and Methods

### General Methods

All biological and chemical reagents were purchased at their highest purity from Sigma Aldrich or Fisher Scientific, unless stated otherwise. pH values of solutions were kept within the ± 0.01 range of the desired pH.

### Sequence Similarity Networks analysis

Sequence similarity networks were constructed in March 2025 using the Enzyme Function Initiative web tools available at https://efi.igb.illinois.edu/ (27, 30, 68). The databases utilized were UniProt and InterPro, versions 2025-01 and 104, respectively. The UniRef90 network was created with the input IPR000310, UniRef90 and the no fragments option, then applying length constraints of a minimum of 400 and a maximum of 2500 residues. Various alignment score thresholds were employed to evaluate the minimal value allowing for separation of enzyme with different substrate specificities in different clusters. For visualization and editing, Cytoscape was used (69).

### Calculation of *dN/dS* ratios

To assess the selection pressure acting on Rv2531c, the *dN/dS* calculation of Rv2531c was performed using the single-likelihood ancestor counting (SLAC) method in the HyPhy software package as described previously (70). A total of 4685 clinical *Mycobacterium tuberculosis* isolates representing major lineages (71–73) were first aligned to the reference genome H37Rv. The genomic region corresponding to Rv2531c was extracted from the resulting whole-genome alignment and translated into amino acid sequences. Codon-based alignments were subsequently generated by combining the nucleotide and amino acid alignments using PAL2NAL. A maximum likelihood phylogenetic tree was constructed from codon-based alignments using IQ-TREE and used as input for SLAC analysis.

### Protein purification

The *rv2531*c gene sequence was amplified from *M. tuberculosis* H37Rv with a N-terminal 6xHis-tag and a TEV cleavage site and ligated into pET-28a(+) plasmid. The plasmid was used to transform into Thermo Scientific™ competent *E. coli* BL21(DE3) cells by heat-shock, and recombinant Rv2531c was over-expressed after overnight induction with 1 mM isopropyl β-D-1-thiogalactopyranoside (IPTG) at OD_600_ of 0.8 and 18 °C in Lysogeny Broth (5 L total volume). Cells were pelleted by centrifugation at 4,300 rpm for 20 minutes and stored at -80 °C until protein purification. On the day of purification, cells were suspended in buffer A [20 mM triethanolamine (TEA), 300 mM NaCl and 20 mM imidazole, pH 7.8] containing lysozyme, cOmplete™ EDTA-free protease inhibitor cocktail, and Invitrogen™ DNase I and were lysed by sonication. The lysate was centrifuged at 15,032 G for 30 minutes at 4°C and the supernatant was used for protein purification. Enzyme was purified by Immobilized Metal Affinity Chromatography (IMAC), using the Cytiva HisTrap™ High Performance 5 mL pre-packed column. About 2 mg/L of enzyme was recovered. The enzyme was purified to homogeneity (> 95% purity), resulting in a single band at 106 kDa as analyzed by SDS-PAGE. Fractions containing Rv2531c were pooled and dialyzed twice in 20 mM TEA, pH 7.8. Protein was then aliquoted, kept at 4 °C and utilized fresh for experiments without loss of activity for over two days. Freeze/thawing led to marked precipitation.

### UV-Vis Spectrometry

The buffer used for the experiment as well as the reference buffer was 20 mM HEPES, pH 7.5. 10 µM Rv2531c was incubated with 10 µM PLP for one hour at 25 °C. The data was acquired on the Shimadzu UV-1900i spectrophotometer. Absorbance from 300-550 nm was collected. Baseline correction was done by subtracting out the absorbance at 550 nm. The data shown are the average of three different independent experiments. The averaged data were analyzed with Origin. The data was smoothed and was deconvoluted for peak detection using a 30 data-point window using the peak deconvolution application in Origin. The data was plotted with Prism.

### Size Exclusion Chromatography

To determine the oligomeric state of Rv2531c, size exclusion chromatography was performed with the Cytiva Superose™ 6 Increase 10/300 GL column. The buffer used was 20 mM sodium phosphate, 150 mM NaCl, pH 7.3. The molecular weight analysis was done according to the manufacturer’s instruction. A calibration curve was constructed with the BIO-RAD Gel Filtration Standard (thyroglobulin(bovine): 670,000 Da; γ-globulin (bovine): 158,000 Da; ovalbumin (chicken): 44,000 Da; myoglobin (horse); 17,000 Da; vitamin B12: 1,350 Da). Linear regression was performed to get the equation:

$$\text{y}=-11.308\times \frac{\left(V_{e}-V_{o}\right)}{\left(V_{t}-Vo\right)}+17.035$$

where *y* is the natural logarithm of the molecular weight of the molecules in Da, *V*_*e*_ is the elution volume of the molecule, *V*_o_ is the void volume and *V*_*t*_ is the bed volume of the column (~24 mL for the column used). The molecular weight for Rv2531c oligomer was back-calculated plugging in the void volume and the elution volume into the above equation. Void volumes and elution volumes were determined using the peak deconvolution application in Origin. The data was plotted with Prism

### Enzyme Function Studies

Rv2531c (2 µM) was incubated with 100 mM substrate (L-lysine, L-ornithine, L-arginine, L-glutamate) in 50 mM sodium phosphate pH 7.3, containing 150 mM NaCl, 10 mM MgCl_2_, 200 µM PLP, 10% D_2_O. Nuclear magnetic resonance (NMR) spectroscopy data were collected on Bruker Advanced 700 MHz and processed with TopSpin v4.3. Kinetics of L-glutamate decarboxylation were followed in 50 mM sodium phosphate, 150 mM NaCl, 10 mM MgCl_2_, 200 µM PLP, 10% D_2_O, pH 7.3. Monosodium L-glutamate concentration varied between 100 and 600 mM. Rv2531c concentration used varied from 100 to 500 nM. Data were collected on Bruker Advanced 700 MHz and analyzed with TopSpin v4.3. The data used for fitting were averaged from the results from three different experiments. Curve fitting was performed with GraphPad Prism. Initial rates were fitted to the Hill equation:

$$v=\frac{V_{max}\times A^{h}}{K_{0.5}^{h}+A^{h}}$$

where *V*_max_ is the maximal velocity, *A* is the substrate concentration, *K*_0.5_ is the substrate concentration at which half the maximal velocity is achieved, and *h* is the Hill number. The Hill number was fixed at 4.

### Microbiological methods

*M. tuberculosis* strains were cultured in Middlebrook 7H9 liquid medium supplemented with 0.5 g/L Fraction V bovine serum albumin, 0.05% tyloxapol and 4% glycerol. For metabolomic profiling studies, *M. tuberculosis* was cultured on Middlebrook 7H10 agar supplemented with 0.5 g/L Fraction V bovine serum albumin, 0.05% tyloxapol, and glycerol according to Brauer et al. (3).

### Construction of deletion and complemented strains

An unmarked in-frame deletion of the *rv2531*c gene was made by amplifying 1.5 kb of flanking sequence from H37Rv with Phusion Taq (New England Biolabs) using specific primers:

upstream of Rv2531c:

Forward: 5’-GCGGATCCGTATTGCCATCGCCT-3’,

Reverse: 5’-GCGAATTCGACGCGTGAAAGCTG-3’;

downstream of Rv2531c:

Forward: 5’-GCGAATTCGTTTGGGTTCATGTC-3’,

Reverse: 5’-GCGGATCCACTGCTGACTTCAAG-3’.

The products were cloned into pCRblunt and sequenced. The inserts were digested with BamH1 and EcoR1 and the fragments were ligated together using T4 DNA ligase. The ligation product was amplified using primers forward and reverse primers and then digested with *BamH*I and cloned into the *BamH*I site of p2NIL. The *PacI* fragment of pGOAL 17 containing the *lacZ* and *sacB* genes were cloned into the *PacI* site of the resulting plasmid to make the Rv2531c KO construct. This was electroporated into H37Rv and single crossovers were selected on Middlebrook 7H11 plates containing kanamycin and X-gal. The blue colonies were then streaked on 7H11 plates and then double crossovers were selected on 7H11 plates containing sucrose and X-gal, the resulting white colonies were screened for double crossovers. The confirmation of the expected deletion was carried out by Southern blot and PCR (not shown).

For the complement, *rv2531*c gene fragment was amplified from Mtb H37Rv genomic DNA by PCR and assembled with pML1335 integrative vector PCR fragment by Gibson assembly (NEB) to create pML1335-Psmyc:*rv2531*c. Competent Mtb:*rv2531*cKO were electroporated with pBS-Int (for integrase) with pML1335-Psmyc:*rv2531*c, and then transferred to 5 mL Middlebrook 7H9 medium with supplement (ADC) and Tyloxapol, and left standing overnight at 37 °C. The electroporated cells were plated on Middlebrook 7H11 medium with supplement (OADC) containing 100 mg/mL Hygromycin B to select for complemented colonies. Complementation was checked by PCR (same primers used in gene amplification noted above) with genomic DNA obtained by InstaGene Matrix (Bio-Rad).

### Metabolomics and stable isotope labeling experiments

*M. tuberculosis* was grown initially in Middlebrook 7H9 liquid media containing the carbon source of interest until the late logarithmic phase and then inoculated onto 0.22 μm nitrocellulose filters under vacuum filtration. *M. tuberculosis*-laden filters were then placed atop chemically equivalent agar media and allowed to grow at 37°C for 5 days. Bacteria were metabolically quenched by plunging Mtb-laden filters into acetonitrile/methanol/H2O (2:2:1, v/v/v) pre-cooled to -40 °C and metabolites were extracted by mechanical lysing of the entire Mtb-containing solution with 0.1 mm Zirconia beads. Lysates were clarified by centrifugation and then filtered with 0.22 μm filters. Bacterial biomass of individual samples was determined by measuring the residual protein content of metabolite extracts. For stable isotope labeling experiments, after 5-day incubation, filter-ladens were transferred to 7H10 plates containing 100% U-^13^C-glycerol or 50% U-^13^C L-glutamate and incubated for 4 hours at 37°C. Metabolites were extracted as described in the previous paragraph. Data shown are mean of three filters (biological replicates) and are representative of two independent experiments.

Metabolite-containing supernatants were directly subjected to LC-MS analysis following the protocol described by Larrouy-Maumus et al. (74). Chromatographic separation was performed using an Agilent 1200 LC system equipped with a solvent degasser, binary pump, temperature-controlled autosampler, and column compartment. A Cogent Diamond Hydride Type C silica column (150 mm × 2.1 mm; dead volume 315 μL) was employed, with a flow rate of 0.4 mL/min. Mobile phases consisted of solvent A (0.2% acetic acid in water) and solvent B (0.2% acetic acid in acetonitrile). The gradient elution profile was Gradient 3 by Pesek et al (69) with modifications: 0–2 min, 85% B; 2–3 min, linear decrease to 80% B; 3–5 min, 80% B; 5–6 min, linear decrease to 75% B; 6–7 min, 75% B; 7–8 min, linear decrease to 70% B; 8–9 min, 70% B; 9–10 min, linear decrease to 50% B; 10–11 min, 50% B; 11–11.1 min, linear decrease to 20% B; 11.1–14 min, hold at 20% B;14.1–18 min, hold at 5% B, followed by a 1 min re-equilibration period at 85% B followed by 6-min stop time. Mass spectrometric detection was carried out using an Agilent Accurate Mass 6230 TOF system. Dynamic mass axis calibration was maintained via continuous infusion of a reference mass solution using an isocratic pump connected to a JetStream ionization source. Data acquisition was performed in both positive and negative modes. Instrument parameters included an ESI capillary voltage of 3500 V, fragmentor voltage of 110 V, nebulizer pressure of 35 psi, nitrogen drying gas flow rate of 13 L/min, and drying gas temperature of 250°C. Spectra were acquired at a rate of 1 spectra/sec over an m/z range of 50–1200. The instrument operated in centroid mode with extended dynamic range (4 GHz), routinely achieving mass accuracy within 5 ppm, resolution between 10,000–25,000 across m/z 121–955, and a dynamic range of up to 10^5^-fold with picomolar sensitivity.

### Construction of mutant enzymes

The following primers from Sigma-Aldrich were used for construction of mutant enzymes:

K406A:

5’-GACCGCAATTGCCACGCCTCGCACCACTACG-3’

5’-CGTAGTGGTGCGAGGCGTGGCAATTGCGGTC-3’

R576A:

5’-CTGTCCGCGCTAGCGCAGGCATCGATG-3’

5’-CATCGATGCCTGCGCTAGCGCGGACAG-3’

R576E:

5’-CTGTCCGCGCTAGAGCAGGCATCGATG-3’

5’-CATCGATGCCTGCTCTAGCGCGGACAG-3’

T606V:

5’-CCCACACCTCGGTTTCGCCCAACCAGC-3’

5’-GCTGGTTGGGCGAAACCGAGGTGTGGG-3’

R718A:

5’-GAACGGGTACGACTTCGCGGAGAAGATCCTGATGG-3’

5’-CCATCAGGATCTTCTCCGCGAAGTCGTACCCGTTC-3’

N731A:

5’-GATTCGGCATCCAGATCGCGAAAACGTCTATCAAC-3’

5’-GTTGATAGACGTTTTCGCGATCTGGATGCCGAATC-3’

The mutants were prepared using the Agilent QuickChange XL Site-Directed Mutagenesis Kit with the original pET-28a(+)*::rv2531c* as the template and plasmids propagated in *E. coli* DH5α Successful construction of the plasmids containing the desired mutants was confirmed by Sanger sequencing, with the pair of primers flanking Rv2531c sequence:

5’-GATCGTCGTGCAGGCC-3’

5’-CGACCAGGTGACGCCG-3’

## Supplementary Material

Fig. S1

## Significance

Enzymes annotated within well-characterized superfamilies are often assumed to have conserved functions, yet this assumption can obscure novel biochemical activities with important biological implications. In this study, we reveal that *Mycobacterium tuberculosis* Rv2531c, previously misannotated as a basic amino acid decarboxylase, is in fact a novel L-glutamate decarboxylase that sustains carbon flux through the GABA shunt. This discovery not only redefines the functional landscape of amino acid decarboxylases but also identifies a previously unrecognized enzyme family conserved across Bacteria, Archaea, and Eukarya. These findings expand our understanding of microbial metabolism and open new avenues for targeting metabolic pathways in pathogenic organisms.
