# Supplementary material for: A cloaked glutamate decarboxylase sustains the GABA shunt in Mycobacterium tuberculosis
Source: Proc Natl Acad Sci U S A. Author manuscript; Available in PMC 2026 Aug 28. (PMC13506015; doi:10.1073/pnas.2619778123)
Supplement: Supplementary file 1 [file EMS217957-supplement-Fig__S1.docx]

**Supporting information**

**A cloaked glutamate decarboxylase sustains the GABA shunt in *Mycobacterium tuberculosis***

H. Minh Thai^#^, Debbie M. Hunt^#^, Yugen Miyahara, Manisha Priya, Htin L. Aung, Remi Zallot and Luiz Pedro S. de Carvalho

**Supporting Figures**

**Figure S1. Full sequence similarity network of Rv2531c homologs in bacteria.** Partial UniRef90 SSN for members of the IPR000310 family, with an alignment score threshold of 185.

**3**

**7**

**4**

**8**

**2**

**1**

**6**

**5**

**Figure S2. Western blot demonstrating deletion of Rv2531c in the Δ*rv2531*c strain.** Lanes 1 and 5 are molecular weight markers. Lanes 2 and 6 are extracts from *M. tuberculosis* H37Rv strain (parent), lanes 3 and 7 are extracts from the Δ*rv2531*c strain and lanes 4 and 8 are extracts from the complemented strain (Δ*rv2531*c::*rv2531*c). Anti-rabbit anti-Rv2531c antibodies were used at 1:10,000 dilution. Image was captured using Li-COR system. Green arrow points to the bands at the expected molecular weight of Rv2531c (~ 106 kDa).

| S. No. | Metabolites | Formula | Molar mass (g/mol) | (M+H)^+^ | RT (min) |
| --- | --- | --- | --- | --- | --- |
| 1 | Putrescine | C_4_H_12_N_2_ | 88.154 | 89.1073 | 16.2 |
| 2 | L-Ornithine | C_5_H_12_N_2_O_2_ | 132.16 | 133.0972 | 13.1 |
| 3 | Agmatine | C_5_H_14_N_4_ | 130.195 | 131.1291 | 16.0 |
| 4 | L-Arginine | C_6_H_14_N_4_O_2_ | 174.204 | 175.119 | 13.5 |
| 5 | Cadaverine | C_5_H_14_N_2_ | 102.181 | 103.123 | 16.5 |
| 6 | L-Lysine | C_6_H_14_N_2_O_2_ | 146.19 | 147.1128 | 13.7 |
| 7 | L-Alanine | C_3_H_7_NO_2_ | 89.094 | 90.055 | 9.6 |
| 8 | β-Alanine | C_3_H_7_NO_2_ | 89.093 | 90.055 | 12.2 |
| 9 | 2-aminobutyric acid (α-ABA) | C_4_H_9_NO_2_ | 103.12 | 104.0706 | 9.2 |
| 10 | 3-aminobutyric acid (β-ABA) | C_4_H_9_NO_2_ | 103.121 | 104.0706 | 11.4 |
| 11 | γ-aminobutyric acid (γ-ABA) | C_4_H_9_NO_2_ | 103.121 | 104.0706 | 12.1 |
| 12 | L-Glutamic acid (Glutamate) | C_5_H_9_NO_4_ | 147.13 | 148.0604 | 8.2 |
| 13 | L-Glutamine | C_5_H_10_N_2_O_3_ | 146.146 | 147.0764 | 10.0 |

**Table S3. Metabolites chromatographic properties.** Chromatographic properties of amino acids, polyamines and isomers are shown which includes m/z and retention times.

**Figure S4. Rv2531c is purified in apo-form.** Spectra shown represent PLP in solution (yellow trace, λ_max_ 389 nm), purified (apo) Rv2531c (black trace), and holo Rv2531c obtained in the presence of additional PLP (red trace, λ_max_ 401 nm, internal aldimine). Each curve is representative of two independent experiments.

**Figure S5. Rv2531c oligomeric state determination.** Size exclusion chromatogram of Rv2531c. The void volume was ~ 8.4 mL. Rv2531c eluted as a symmetrical peak, at 14.1 mL. Based on standards, the apparent molecular weight was calculated to be around 405 kDa, which suggests that Rv2531c is a tetramer in solution. For reference, the molecular weight for monomeric Rv2531c is ~ 106 kDa. The data shown are representative of three different experiments.

**Figure S6. Rv2531c requires Mg^2+^ for L-glutamate decarboxylation.** ^1^H NMR spectra of Rv2531c and L-glutamate, in the presence or absence of Mg^2+^. Upon incubation of Rv2531c with L-glutamate (in the absence of Mg^2+^**)** for 1 hour, there were no new peaks in the spectra. In the presence of Mg^2+^, three new peaks corresponding to the protons of GABA appeared in the spectrum.

A

B

C

D

**Figure S7. Active site of Rv2531c.** (**A**) Overview of the active site of Rv2531c with the modelled L-α-hydrazino acid L-glutamate analog (green) using the L-α-hydrazino acid L-lysine analog from 9E0O. (**B**) Possible catalytic residues in close proximity to the modelled L-α-hydrazino acid L-glutamate analog. Chain A of the PLP-loaded Rv2531c dimer is colored orange, and T606 from chain B is colored yellow. (**C**) UV-Vis spectra of parent Rv2531c in apo and holo forms and free PLP. (**D**) UV UV-Vis spectra of Rv2531c K406A in apo and holo forms and free PLP. 5 µM of PLP and 5 μM of Rv2531c K406A were used. There is no shift in peak for Rv2531c K406A. Each curve is representative of two independent experiments.
